# Revolutionizing ovarian cancer treatment: cryptic antigens as the next breakthrough in immunotherapy

**DOI:** 10.1097/MS9.0000000000003372

**Published:** 2025-05-20

**Authors:** Nisha Khatri, Harendra Kumar, Bharat Kumar

**Affiliations:** a Liaquat University of Medical and Health sciences, Jamshoro, Pakistan; b Dow University of Health Sciences, Karachi, Pakistan; c Nepal Medical College, Kathmandu, Nepal

Ovarian cancer is a serious health problem, ranking as the seventh most frequent malignancy and the eighth main cause of cancer-related deaths among women worldwide. This illness is divided into three basic subgroups depending on its cellular origin, with the epithelial subtype being the most frequent and accounting for the majority of cases^[^1^]^. Global data have revealed 314 000 new cases and 20 700 fatalities recorded in 2020, with the greatest incidence rates seen in European nations with a high Human Development Index, thus projecting a large increase in cases in the future, creating a severe health problem^[^2^]^. Treating ovarian cancer remains a hefty issue since the tumor has a bad prognosis, and it is already at an advanced stage when discovered because diagnosing ovarian cancer at early stages is difficult owing to its non-specific appearance. Additionally, the screening approaches like ultrasound imaging and serum biomarkers are not accurate to observe abnormal characteristics for its early identification. A new study has revealed a new targeted therapeutic for ovarian cancer, altering treatment choices for patients. A recent research study undertaken at the immunology department of the Mayo Clinic found 311 distinct cryptic antigens from non-coding regions of DNA in tumor cells .The research indicated that cryptic antigens are the primary immunogenic proteins in ovarian cancer, which may stimulate peptide-specific T-cells; consequently, they can be employed as targeted immunotherapy for treating this malignancy^[^3^]^.

Cryptic antigens, or cryptic epitopes, are concealed peptides that are unavailable to immune cells for detection, enabling auto reactive T-cells to avoid elimination throughout immunological development. There are many processes that are responsible for this concealing of these epitopes, such as anatomical sequestration, degradation by proteolytic enzymes before presenting to MHC molecules, poor binding affinity to MHC class II molecules, or inadequate concentration of epitopes^[^4^]^. Cryptic epitopes play a vital role in autoimmune disorders. During inflammation, tissue injury, or infection, higher quantities of cryptic epitopes attach to Major Histocompatibility Complex (MHC) molecules and are identified as foreign antigens by T cells, thereby activating an immune response^[^4^]^. In the setting of cancer, cryptic antigens become immunogenic by aberrant translation processes such as alternative open reading frames or translation of non-coding sequences that lead to the presentation of new peptides on tumor cells that are recognized by the immune system. Neoantigens, however, vary from these atypical cryptic antigens, since they emerge primarily from neoplastic cells via somatic mutations such as single nucleotide variations, insertions, or deletions, which result in unique peptide synthesis and detection by T cells^[^5,6^]^.

Many medicines have shown an initial response to treat ovarian cancer. Immune check point inhibitors (ICIs), such as programmed cell death protein 1 inhibitors (Nivolumab, Pembrolizumab, etc.) and cytotoxic T lymphocyte-associated protein 4 inhibitors (Ipilimumab), have shown success in many cancers, but their response rate in ovarian cancer patients is still low. Furthermore, the use of ICIs is linked with several immune-related side consequences^[^7,8^]^. Neoantigens have been examined as targets for tailored cancer immunotherapy. However, low mutation load and restricted neoantigen presentation associated with ovarian cancer have hampered the efficacy of neoantigen-targeted vaccines^[^9^]^, highlighting the need for new immunotherapeutic methods targeting overexpressed antigens. Ovarian cancer demonstrates tumor heterogeneity and an immunosuppressive microenvironment, making immunotherapy less effective^[^9,10^]^. These results underline the necessity for identifying tumor-specific traits, which are typically disregarded, and the urgent need for viable alternative multicomponent and new treatments.

A major advance in cancer immunotherapy is the revelation that cryptic antigens are immunogenic targets in ovarian cancer^[^6^]^. To increase the immune system’s potential to recognize and destroy tumor cells, researchers are currently looking at techniques to build cryptic antigen-based treatments, such as customized cancer vaccines and T-cell-based therapeutics^[^7^]^. The objective of continuing research is to expand the utilization of cryptic antigen presentation mechanisms in adoptive cell therapy, notably via modified T cells that are programmed to recognize these unique epitopes.

Beyond ovarian cancer, cryptic antigen-based techniques hold potential for other illnesses where typical immunotherapies have failed because of tumor heterogeneity and immune evasion, such as lung, pancreatic, and colorectal malignancies. The paradigms of cancer treatment may evolve if research efforts to uncover and verify cryptic antigens across cancer types are increased.

Sustained funding for research and clinical trials is essential to turning these discoveries into real-world applications. A thorough analysis of the safety, effectiveness, and long-term advantages of cryptic antigen-targeted drugs will be necessary to ensure their successful implementation. To expedite the development of new immunotherapeutics, researchers, clinicians, and industry executives must collaborate to close the gap between laboratory findings and patient treatment. The discovery of cryptic antigens in ovarian cancer highlights the pressing need for lawmakers, academics, and the medical community to address funding and research for cryptic antigen-based immunotherapies (Table 1). If we encourage these findings, we may be able to accelerate the development of new cancer drugs that work better and are less harmful than current therapies. Activism and public awareness are essential to generating support for ovarian cancer research. More funding has to be allocated to clinical trials and translational research if potential immunotherapies are to reach patients in a timely manner. Collaboration between academic institutions, pharmaceutical companies, and government agencies may also hasten the regulatory approval process and provide access to cutting-edge therapies. Since cryptic antigens are opening the door to more individualized and effective cancer therapies, immunotherapy has a bright future. Supporting ongoing research, funding, and public involvement might transform cancer treatment and provide hope to millions of people worldwide.

**Table 1:** Future implications of cryptic antigen-based immunotherapy in ovarian cancer

Research area	Key advancements	Potential impact
Cryptic antigen-based therapies	Development of vaccines and T-cell-based treatments targeting cryptic antigens.	Enhances immune response, improving treatment specificity.
Application to other cancers	Exploration of cryptic antigen expression in other malignancies (e.g., breast, lung, and pancreatic cancer).	Broadens the scope of immunotherapy beyond ovarian cancer.
Personalized medicine	Identification of patient-specific cryptic antigens using advanced sequencing technologies.	Enables individualized cancer treatment strategies.
Clinical trials & translation	Ongoing trials assessing the safety and efficacy of cryptic antigen-based immunotherapies.	Bridges the gap between research and patient care.
Challenges & future directions	Overcoming tumor heterogeneity and immune evasion mechanisms.	Optimizing immunotherapy effectiveness in diverse patient populations.

## Data Availability

The data that support the findings of this study are available from the corresponding author upon reasonable request.
